# A rare case of cutaneous Langerhans cell histiocytosis in an adult patient

**DOI:** 10.1016/j.jdcr.2023.12.023

**Published:** 2024-01-20

**Authors:** Aleena Boby, Nirav Shah, Ann Lin

**Affiliations:** a Morsani College of Medicine, University of South Florida, Tampa, Florida; b Department of Dermatology and Cutaneous Surgery, USF Health Morsani College of Medicine, Tampa, Florida

**Keywords:** Langerhans cell histiocytosis

*To the Editor:* Kuo et al^1^ recently published a case of cutaneous only Langerhans cell histiocytosis (LCH) in an adult. We are aware of very few published cases of this clinical presentation. Here, we describe one such case in a 62-year-old adult male.

A 62-year-old man with a history of intellectual disability presented to our clinic with a progressively diffuse pruritic rash that started 3 years prior. Despite previous treatments for allergic contact dermatitis and tinea corporis using topical corticosteroids and antifungals, the rash remained unimproved. Physical examination revealed circular follicular accentuated erythematous plaques and erythematous annular plaques on the scalp, chest, upper arms, dorsal hands, abdomen, inguinal folds, buttocks, and bilateral thighs (Fig 1). Since the patient was nonverbal, his sister and caregiver acted as the historian. Subsequently, biopsies were performed at 2 different sites due to concerns for cutaneous T-cell lymphoma (Fig 2).

**Fig 1:**
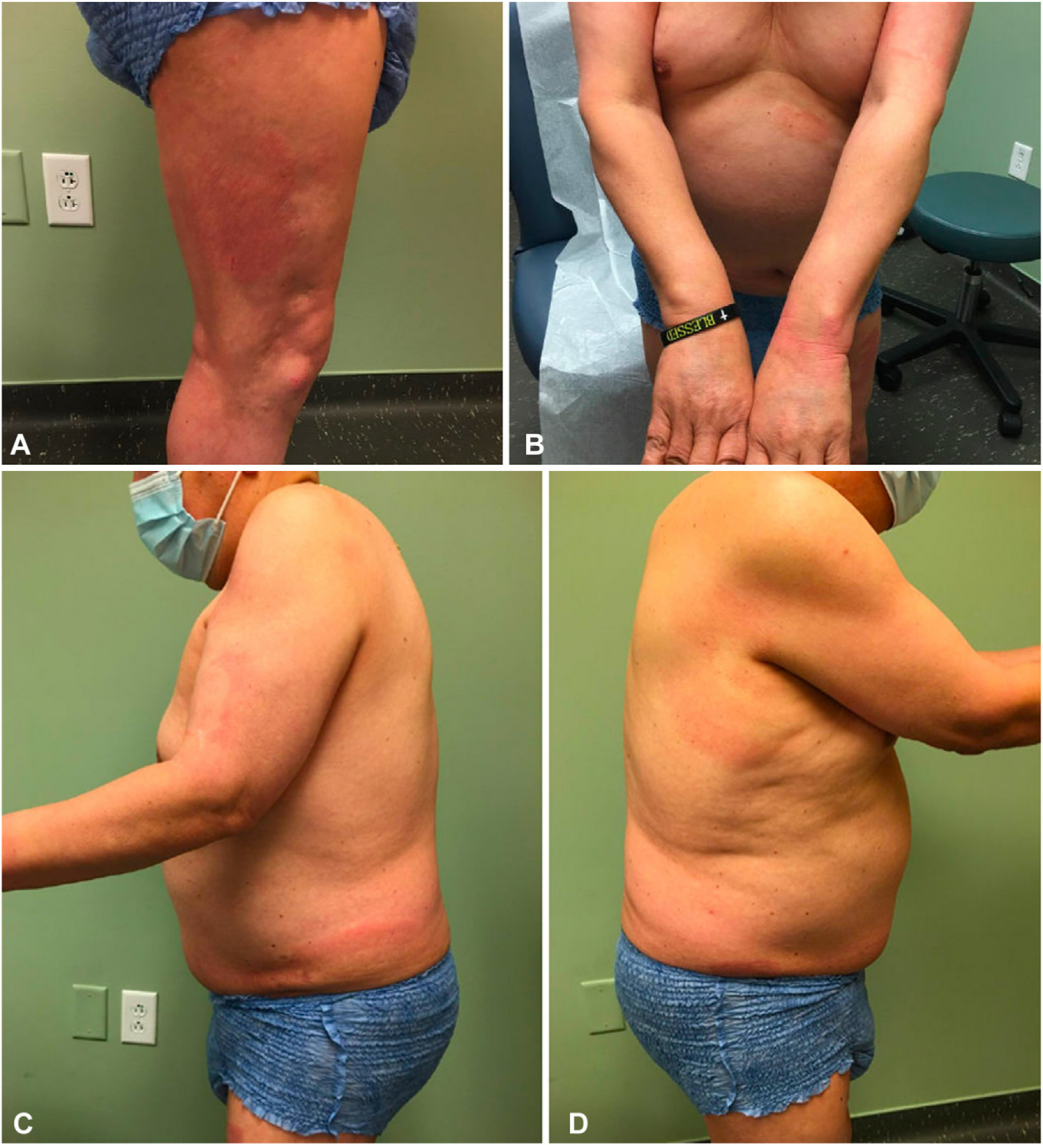
Initial visit. **A,** Right leg, (**B**) torso and upper extremities, (**C**) left lateral torso/arm, and (**D**) right lateral torso/arm.

**Fig 2:**
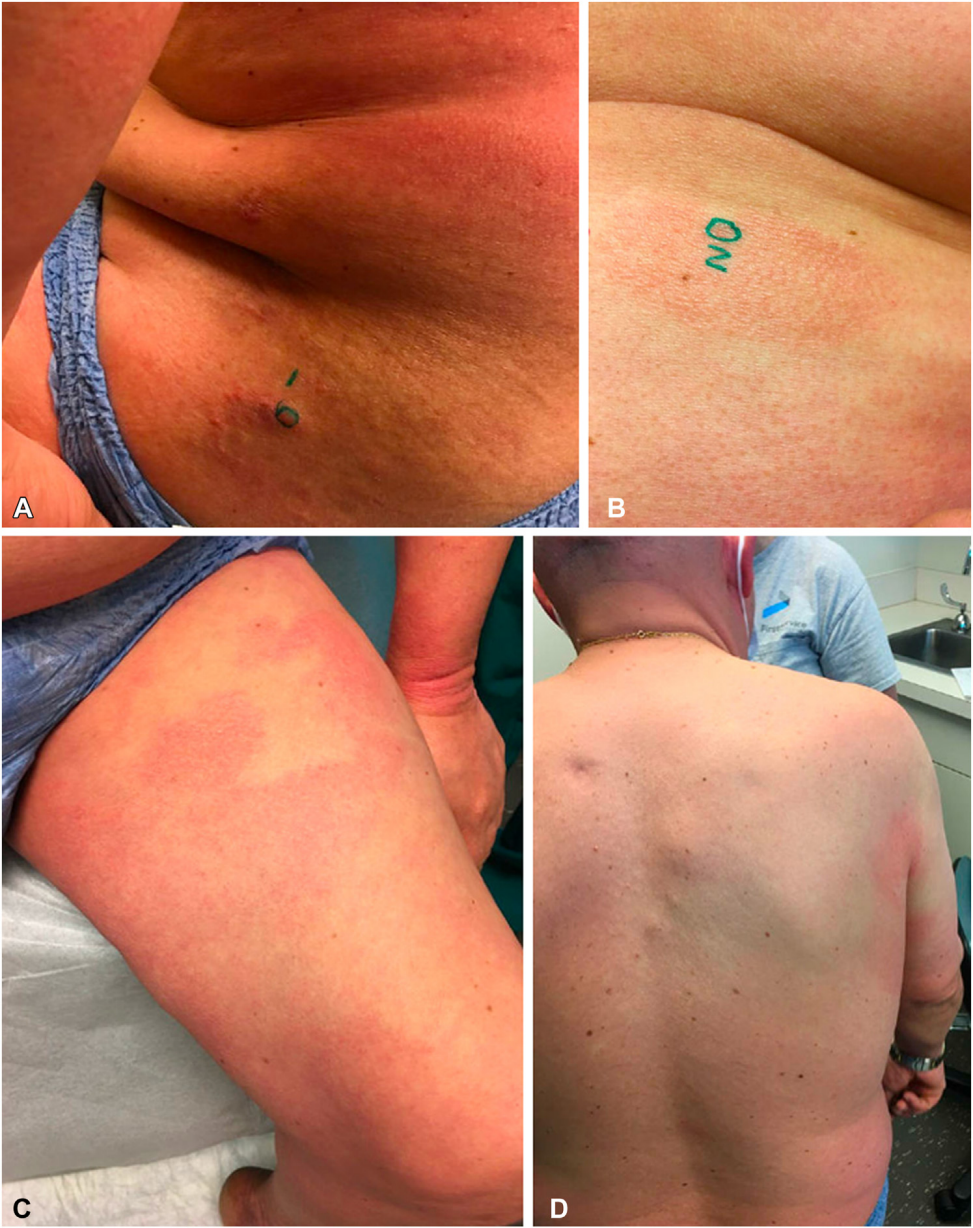
Second visit 1 Month later. **A,** Left hip (biopsy site), (**B**) left abdomen (biopsy site), (**C**) left anterior thigh, and (**D**) back.

Dermatopathology findings indicated a dense inflammatory infiltrate within the dermis, extending focally around deep vessels (Fig 3). Additionally, an increased number of lymphocytes, histiocytes, neutrophils, eosinophils, and numerous “bean-shaped” histiocytoid cells were noted. These cells stained positive for S-100 and CD1a, while CD-3 and CD-20 stains demonstrated a mixture of T and B cells. A CD-30 stain highlighted rare cells within the infiltrate. Collectively, these findings supported a diagnosis of LCH.

**Fig 3:**
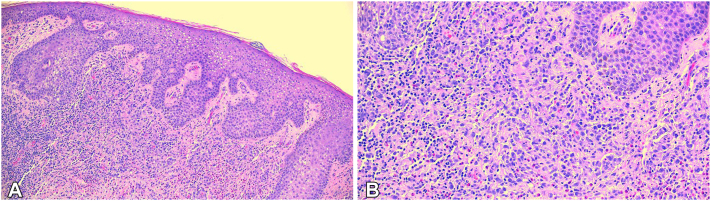
Pathology results. **A,** View of inflammatory infiltrate into layers of the dermis. **B,** Closer view.

The patient was referred to Moffitt Cancer Center for further evaluation of possible systemic involvement. Bone marrow biopsy revealed a normocellular marrow. Positron emission tomography-computed tomography scan showed no abnormalities. Blood work, including pituitary hormonal studies, showed slightly elevated adrenocorticotropic hormone levels but was otherwise unremarkable. Magnetic resonance imaging of the brain revealed a 3 × 2 cm mass in the fourth ventricle, which could not be surgically resected due to anatomical limitations. However, cerebrospinal fluid analysis showed no evidence of metastatic disease, thereby suggesting a benign etiology such as a subependymoma or choroid plexus papilloma.

LCH is a rare lymphoproliferative disease that is characterized by the presence of langerin-positive (CD207+) dendritic cells with a high affinity for bone, skin, lung, and the pituitary gland.^2^ This disease primarily affects children, with a male-to-female ratio of 1.2:1.^3^ The clinical presentation of LCH varies widely, leading to diverse treatment approaches based on disease severity and extent of organ involvement.^2^^,^^4^ Since it mainly presents in children, there are no standard therapies for LCH in adults.^4^ Treatment for multifocal disease typically involves chemotherapy, specifically vinblastine, combined with prednisone.^2^ Surgical excision is considered in cases of isolated cutaneous disease.^4^

This case highlights a rare occurrence of adult-onset LCH, with an estimated incidence of 1 to 2 per million adults.^5^ Systemic evaluation predominantly yielded negative results, indicating isolated cutaneous LCH. There are few reported cases of cutaneous only adult-onset LCH. In fact, cutaneous disease, including those with organ involvement, is seen in only 33% of all cases.^6^ Given these findings, we predict a good prognosis, with the use of a combination of topical corticosteroids, narrowband UV-B phototherapy, and systemic treatments (ie, low-dose methotrexate) for disease improvement and symptom control.

## Conflicts of interest

None disclosed.
